# Investigating the Effect of Lumbar Spinal Stenosis (LSS) Surgery on Sexual Function in Male Patients over 50 Years

**DOI:** 10.3390/medicina61040628

**Published:** 2025-03-29

**Authors:** Reza Fatahian, Saeed Gharooee Ahangar, Mehran Bahrami Bukani, Masoud Sadeghi, Annette B. Brühl, Serge Brand

**Affiliations:** 1 Department of Neurosurgery, Kermanshah University of Medical Sciences, Kermanshah 6714415333, Iran; rfatahian@gmail.com (R.F.); saied_gharooee@yahoo.com (S.G.A.); 2 Students Research Committee, Kermanshah University of Medical Sciences, Kermanshah 6714869914, Iran; mehranbahrami69@gmail.com; 3 Medical Biology Research Center, Health Technology Institute, Kermanshah University of Medical Sciences, Kermanshah 671551616, Iran; sadeghi_mbrc@yahoo.com; 4 Center for Affective, Stress and Sleep Disorders, Psychiatric Clinics, University of Basel, 4002 Basel, Switzerland; annette.bruehl@upk.ch; 5 Sleep Disorders Research Center, Kermanshah University of Medical Sciences, Kermanshah 6714869914, Iran; 6 Substance Abuse Prevention Research Center, Kermanshah University of Medical Sciences, Kermanshah 6714869914, Iran; 7 Division of Sport Science and Psychosocial Health, Department of Sport, Exercise and Health, University of Basel, 4031 Basel, Switzerland; 8 School of Medicine, Tehran University of Medical Sciences, Tehran 1339973111, Iran; 9 Center for Disaster Psychiatry and Disaster Psychology, Center of Competence of Disaster Medicine of the Swiss Armed Forces, Psychiatric Clinics, University of Basel, 4002 Basel, Switzerland

**Keywords:** lumbar spinal stenosis, surgery, erectile function, sexual function, Iran

## Abstract

*Background and Objectives*: Lumbar spinal stenosis (LSS) is a leading cause of back surgery in elderly individuals. Additionally, LSS can result in buttock pain; abnormal sensations; or even loss of sensation in the thighs, feet, legs, and buttocks, as well as potential loss of bowel and bladder control. As a further consequence, sexual activity is impaired. However, there is limited information on sexual function in patients undergoing LSS surgery, in general, and among male patients, in specific. Accordingly, the aim of this study was to investigate the effect of LSS surgery on sexual function in male patients over 50 years. *Materials and Methods:* Participants were fifty male patients with LSS aged 50 years and older who underwent LSS surgery at the Imam Reza Hospital in Kermanshah from March 2024 to the end of 2024. To assess sexual performance over time, participants completed the International Index of Erectile Function (IIEF-15) questionnaire both before LSS surgery and six months after LSS surgery. For pre–post comparison, we used paired *t*-tests. *Results*: Compared to the pre-surgery stage, six-month post-surgery improvements were erectile function (+21%; Cohen’s d: 1.40), orgasmic function (+35.1%; Cohen’s d: 1.49), sexual desire (+27.3%; Cohen’s d: 1.48), intercourse satisfaction (+14% Cohen’s d: 0.77), overall satisfaction (+34.6% Cohen’s d: 1.74), and overall sexual function (+25.3%; Cohen’s d: 1.48). *Conclusions*: Among a sample of male patients aged 50 years and older, LSS surgery improved all dimensions of sexual satisfaction, including orgasmic, erectile, and sexual functions; sexual desire; intercourse satisfaction; and overall satisfaction. Medical doctors treating males with LSS might consider informing their patients about the favorable effects of LSS surgery on sexual life and sexual satisfaction.

## 1. Introduction

Lumbar spinal stenosis (LSS) impacts around 103 million individuals globally and affects 11% of older adults in the United States [1]. The primary treatment includes activity modification, pain management, and physical therapy, while the long-term effectiveness of epidural steroid injections remains unproven [1]. LSS causes significant pain and disability and is the leading reason for spinal surgery in patients over 65 [2]. Typically, individuals with LSS report pain in buttocks; abnormal sensations, including the absence of sensations in the legs, thighs, feet, buttocks, or even the loss of bladder and bowel control [1]. As a further consequence, sexual activity is impaired. Further, LSS is a complex clinical syndrome caused mainly by degenerative changes, and LSS is usually diagnosed in individuals with symptoms related to lumbar spinal canal narrowing seen on imaging studies [3]. However, and against expectations, a significant correlation between radiological findings and the severity of clinical symptoms was scarcely observed [4]. As such, experts still do rely on a patient’s self-reported perception of pain and impairments, including LSS-related sexual issues.

Additionally, the effectiveness of LSS treatment relies on the accuracy of its diagnosis, which can be challenging. Since no universal gold standard for LSS diagnosis exists, clinical diagnoses often depend on the judgment of expert clinicians [5]. If conservative treatment fails to improve symptoms within three to six months, surgical treatment may be considered as an option to relieve symptoms [6]. Surgery provides better outcomes for at least four years in terms of disability and pain, with decreasing benefits over time compared to conservative treatment [6,7]. For patients with LSS and additional health conditions (especially diabetes), it is important to consider a higher risk for adverse events when making treatment decisions. Advanced age by itself was not linked to an increased risk for adverse events, less functional improvement, less symptomatic relief, or reduced treatment satisfaction [8].

Generally, most patients experienced pain relief within three months, and some even improved earlier, while others took up to a year. After four years, approximately 50% of patients undergoing conservative treatment reported excellent or fair outcomes, compared to about 80% of those who underwent surgery [9].

LSS can significantly impact a patient’s quality of life and functional abilities, including sexual health, which plays a vital role in physiological well-being and relationships [10,11,12]. However, sexual dysfunction in spinal patients and sexual health counseling are not often performed in neurosurgical care, and there is limited information on sexual function and health in patients undergoing surgery for LSS. Therefore, the aim of this study was to investigate the impact of LSS surgery on sexual function in male patients over 50 years of age.

One might wonder why sexual function, though not life-threatening, is a significant concern. In humans, sexual activity and intimacy serve at least five distinct purposes: (1) exploring a partner’s values, (2) reproduction, (3) fostering pair-bonding and relationship stability [13,14], (4) joy [15,16,17], and (5) quality of life [15,18,19,20,21,22,23]. The sexual activity of (heterosexual) couples usually signifies exclusivity, intimacy, and bond-reinforcing behavior [17]. Further, while typically private, the sexual activity and sexual intercourse of heterosexual couples can occur under many different conditions: (1) before and after the female’s fertile phase (ovulation), (2) during pregnancy and (3) during the female’s post-menopausal stage, thus indicating that, for heterosexual couples, sexual intercourse fulfills needs beyond reproduction. Given its exclusivity and significance for bonding and relationship quality, its impairment can be deeply distressing for both individuals and couples. Research has consistently shown a strong connection between sexual satisfaction and overall life satisfaction [15,18,19,20,21,22,23,24].

Given this background, we hypothesized that LSS surgery positively impacted on sexual function in male patients over 50 years of age. Further, considering the existing gap in research, a further scientific question was, if and if so, to what extent LSS surgery might have favorably impacted on patients’ sexual satisfaction and overall sexual quality of life. With this, the present study had the potential to provide a more comprehensive understanding of the long-term benefits of LSS surgery, beyond mere physical health. By diving deeper into these areas, the study could offer insights into both the physiological and emotional aspects of recovery, helping to improve patient care and outcomes.

## 2. Material and Methods

### 2.1. Procedure

Male patients with LSS and who were ready to undergo LSS surgery at the Imam Reza Hospital in Kermanshah (Iran) were approached from March 2024 to the end of 2024 to participate in the present study. They were thoroughly informed about the study’s objectives and the confidential, anonymized handling of their data. Subsequently, they provided written informed consent. Two to three days before and six months after surgery, participants completed a brief visual analogue scale to indicate pain intensity and a questionnaire to assess sexual function (see below). The local ethical committee approved the study (registration code: IR.KUMS.MED.REC.1403.051), which was performed in accordance with the seventh and current version [25] of the Declaration of Helsinki, Research of Kermanshah University of Medical Sciences. The study was implemented as follows: informed consent was obtained from the patients, and before the surgery, patients were given the questionnaire (https://www.browardurologycenter.com/pdf/International-Index-of-Erectile-Function-IIEF-Questionnaire.pdf) (access date: 12 March 2024).

### 2.2. Participants

Inclusion criteria were (1) male gender; (2) age 50 years or older; (3) suffering from LSS, as ascertained by an experienced medical doctor after a thorough medical examination and clinical interview; (4) willing and capable of adhering to the study requirements; (5) stable and emotionally satisfying couple relationship; and (6) signed written informed consent. Exclusion criteria were (1) withdrawing from the study; (2) reporting further comorbidities, such as diabetes, cardiovascular, and musculoskeletal diseases; and (3) undergoing further surgeries.

Of the 75 individuals approached for the study, 18 (24%) had cervical stenosis surgery, and 7 (9.3%) underwent both lumbar and cervical stenosis surgery; the final sample was 50 (66.6%).

We clarify that all patients in our study were operated on by the same surgeon, ensuring consistency in surgical techniques. Magnetic resonance imaging confirmed a clear lumbar block in one or more segments for all included patients.

### 2.3. Measures

#### 2.3.1. Sociodemographic Characteristics

Participants reported on their sex (male), age (years), age of onset of backpain (years), education (illiteracy or low literacy; diploma and postgraduate; bachelor degree or higher), current job position (employed, unemployed, freelancer, job with low or heavy physical efforts, farmer, military cadre, or retired), place of residence (urban vs. non-urban area), tobacco use, alcohol use, hookah use, and other drug use (yes vs. no) (see Table 1). Pain was measured using a 0–10 scale, where 0 represents no pain and 10 represents the worst pain imaginable. Relationship satisfaction was assessed using a 5-point scale, ranging from 1 (low satisfaction) to 5 (high satisfaction).

#### 2.3.2. Sexual Function

Participants completed the Farsi/Persian version [26,27,28,29] of the International Index of Erectile Function (IIEF-15) [30,31]. The IIEF comprises 15 items that evaluate sexual desire, orgasmic function, erectile function, intercourse satisfaction, and overall satisfaction. An example of a typical item is as follows: “How often were you able to get an erection during sexual activity?” And the response options are as follows: 0 (no sexual activity), 1 (almost never), 2 (a few times (much less than half the time)), 3 (sometimes (about half the time)), 4 (most times (much more than half the time)), and 5 (almost always/always). While the labels for anchor points differ depending on the specific question, a higher score always indicates less erectile dysfunction. Consequently, a higher sum score reflects lower erectile dysfunction and, thus, better erectile function (Cronbach’s alpha = 0.90).

#### 2.3.3. Analytic Plan

Sociodemographic information was reported in number and frequency (%). With a series of paired *t*-tests, we compared the pre- and post-surgery means and standard deviations of dimensions of sexual functions. The level significance was set at 0.05. Effect sizes for t-tests were reported as Cohen’s d with the following cut-off points [32,33,34]: d < 0.49 (small effect size); 0.50 < d < 0.79 (medium effect size); and d > 0.80 (large effect size). Data analysis was performed with SPSS^®^ version 25 (IBM Corporation, Armonk, NY, USA).

## 3. Result

### 3.1. Sociodemographic Information

The age distribution of the 50 participants was as follows: 12 patients (24%) aged 50–60, 27 patients (54%) aged 60–70, and 11 patients (22%) over 70. The age of onset of back pain varied: 8 patients (16%) between 20 and 30 years, 19 patients (38%) between 30 and 40 years, and 23 patients (46%) between 40 and 50 years. Regarding education, 34 patients (68%) were illiterate or poorly educated, 12 patients (24%) had a diploma or post-diploma degree, and 4 patients (8%) had a bachelor’s degree or higher. Employment status included 5 patients (1%) unemployed, 2 patients (4%) self-employed, 4 patients (8%) unemployed, 8 patients (16%) in military service, 15 patients (30%) with heavy physical jobs, 8 patients (16%) with low physical jobs, 4 patients (8%) employees, 3 patients (6%) retired, and 9 patients (18%) who were farmers. Of the 50 patients, 17 (34%) lived in urban areas and 33 (66%) in rural areas. Regarding smoking habits, 39 patients (78%) were smokers, and 11 (22%) were non-smokers. Hookah-use history showed 28 patients (56%) had used hookah, while 22 (44%) had not. Alcohol-consumption history revealed that 31 patients (62%) had consumed alcohol, whereas 19 (38%) had not. In terms of drug use, 27 patients (54%) were positive, and 23 (46%) had no history of drug use (Table 1).

### 3.2. Sexual Function over Time

Table 2 reports the change in performance (percentages) regarding sexual functions, and Table 3 reports the descriptive and inferential statistical indices of sexual function, body mass index (BMI), pain, and relationship satisfaction, both three days before and six months after SLL surgery. For changes in performance, improvements were observed in all dimensions (Table 2).

## 4. Discussion

The aims of the present study were to investigate, among a sample of 50 males aged 50 years and older, if undergoing LSS surgery impacted on participants’ sexual functions. Results showed, compared to the state before LSS surgery, six months after surgery, sexual functions increased, in general; and in all subdimensions, such as erectile function, intercourse satisfaction, or overall sexual satisfaction, specifically, increased as well. The present study adds to the literature in the following important ways. First, to our understanding, this is the first evidence-based study to show that LSS surgery appears to have a direct favorable impact on males’ sexual functioning. Second, all participants showed consistent improvements, irrespective of their sociodemographic background. Overall, the findings suggest that LSS surgery can substantially enhance sexual health and quality of life in older male patients, underscoring the importance of considering sexual health outcomes in treatment plans for LSS.

At a broader level, diagnosing LSS in elderly patients can be challenging due to the wide range of presentation nuances and common comorbidities, such as degenerative disc disease. Treatment options vary from conservative to surgical, with surgery typically reserved for cases involving additional neurological issues or when conservative measures have proven ineffective [5]. To illustrate the point, patients with LSS without degenerative spondylolisthesis who underwent surgery experienced significantly greater improvements in pain, function, satisfaction, and self-rated progress compared to those who received nonsurgical treatment [35]. However, there is no consensus regarding the best treatment of patients with LSS [36,37], and decisions in favor of conservative or surgical treatments appear to be highly individualized and ad hoc decisions.

Further, we note that the literature on SLL and SLL-related surgeries in relation to an individual’s sexual activity is scarce. One study [38] investigated the characteristics of female sexual dysfunction related to spinal pathology and surgery. The authors concluded that female sexual dysfunction from the spine is caused by traumatic causes, malignant tumors, and benign tumors with and without bone involvement. They also found a significant reduction in sexual pain after surgery, but [38] noted that other types of sexual dysfunction not associated with sexual pain do not improve after surgical intervention. Another study [39] examined the presence of erectile dysfunction in both cervical and lumbar stenosis groups and found no significant association with any identified risk factors. Wottrich, Kha, Thompson, Bakar, Yee, Melillo, Nash, Healy, Steinmetz and Mroz [39] concluded that there was no significant improvement in overall erectile function postoperatively for patients who had erectile dysfunction before the surgery. Clearly, our data do not support Wottrich et al.’s findings.

Holmberg, Vangen-Lønne, Gulati, Nygaard, Solberg, Salvesen and Gulati [12] examined changes in pain during sexual activity after LSS surgery. They concluded that a large proportion of patients undergoing LSS surgery experienced improvement in pain during sexual activity at one year. Malik, et al. [40] conducted a systematic review on the impact of spinal surgery on sexual function and found that anterior lumbar approaches were linked to a higher incidence of retrograde ejaculation, particularly when the transperitoneal laparoscopic approach was used. Malik, Jain, Kim, Khan and Yu [40] also found that there was inconclusive evidence regarding a bodily position during the sexual activity. They concluded that, despite limited high-quality evidence, there was a general trend toward improved sexual function following spinal surgery, and Malik, Jain, Kim, Khan and Yu [40] suggested that future studies use the assessment of sexual function as an important determinant of quality of life to guide appropriate preoperative counseling. Last, one study [41] investigated the effect of LSS and surgical decompression on erectile function. The authors observed a significant reduction in severe preoperative back and leg pain following decompression surgery, which correlated with notable improvements in sexual quality of life. However, the incidence of erectile dysfunction remained higher both before and after surgery compared to population-based data, with a significant decline in erectile function noted at the final follow-up seven to nine months post-surgery.

Despite the novelty of the present research, this study had the following limitations: First, the sample was highly selective (males aged 50 years and higher); as such, results should not be transferred to younger males with LSS, and not to females, irrespective of their age. Second, our study included a relatively small sample, and at the same time, no control sample was assessed, which may limit the generalizability of our findings to broader populations. Third, inclusion criteria were such that exclusively individuals with LSS were assessed, while clinical reality shows that adults with LSS often suffer from further comorbidities, such as diabetes, psychiatric issues, cardiovascular, and musculoskeletal diseases [8]. Fourth, patients reported on their current pain intensity and limited mobility both pre- and post-surgery, and, sadly, such assessments took place in a rather informal fashion during the medical and clinical interviews. As such, it would have been clinically and medically interesting to know if post-surgery improvements in sexual satisfaction were also related to improved pain and mobility. Future studies should consider assessing both mobility and pain systematically and thoroughly.

## 5. Conclusions

The findings suggest a significant enhancement in sexual desire, orgasmic function, erectile function, intercourse satisfaction, overall satisfaction, and overall sexual function following LSS surgery in men aged 50 and older. Collectively, these improvements highlight the positive impact of LSS surgery on the sexual health and quality of life of older male patients. This study also highlights the need for healthcare providers to address sexual health concerns as part of the overall management plan for patients undergoing LSS surgery.

In addition to these clinical improvements, the results underscore the importance of a holistic approach to treating patients with LSS. Future research should aim to incorporate larger sample sizes; control groups; and considerations of potential confounding factors, such as comorbidities and medication use, to further substantiate the findings. Furthermore, clinicians should be aware of the potential influence of psychological and neurological factors on sexual function and engage in preoperative discussions regarding the possibility of sexual health improvements. These findings, though promising, also warrant further exploration within the wider research landscape of spinal surgery and sexual health outcomes. By emphasizing the practical implications for both healthcare providers and patients, this study underscores the necessity of a comprehensive care plan that includes attention to sexual health following spinal surgery.

## Figures and Tables

**Table 1:** Results of the frequency and percentage of demographic characteristics of male patients with lumbar spinal stenosis over 50 years of age.

Variable	Number (%)
Age range (years)	
50–60	12 (24)
60–70	27 (54)
Above 70	11 (22)
Age of the onset of back pain (years)	
20–30	8 (16)
30–40	19 (38)
40–50	23 (46)
Education	
Illiteracy and low literacy	34 (68)
Diploma and postgraduate	12 (24)
Bachelor’s degree and higher	4 (8)
Job	
Unemployed	5 (1)
Job free	2 (4)
Military	4 (8)
Job with activities heavy physical	15 (30)
Job with activities low physical	8 (16)
Employee	4 (8)
Retired	3 (6)
Farmer	9 (18)
Place of residence	
Urban	17 (34)
Rural	33 (66)
Smoking	
Yes	39 (78)
No	11 (22)
Hookah use	
Yes	28 (56)
No	22 (44)
Alcohol consumption	
Yes	31 (62)
No	19 (38)
Drug use	
Yes	27 (54)
No	23 (46)
Level of the stenosis	
L4-L5	25 (50)
L3-L4 and L4-L5	15 (30)
L3-L4, L4-L5, and L2-L3	10 (20)

**Table 2:** Results of percentage of medical and clinical records in patients with lumbar spinal stenosis in men aged 50 years and older.

Variable	Performance Percentage Before Surgery	Performance Percentage After Surgery	Difference
Erectile function	50.0	71.0	+21.0
Orgasmic function	47.5	82.6	+35.1
Sexual desire	47.5	74.8	+27.3
Intercourse satisfaction	57.0	71.0	+14.0
Overall satisfaction	44.6	79.2	+34.6
Sexual function	49.9	74.4	+24.5

**Table 3:** Effect of lumbar spinal stenosis surgery on pain and sexual function.

Dimension	Before Surgery (Mean; SD)	After Surgery (Mean; SD)	Difference (Mean; SD)	t	Cohen’s d
Erectile function	15.09 (4.22)	21.45 (4.79)	6.36 (4.67)	t(49) = 10.11 ***	1.40 (L)
Orgasmic function	4.71 (2.31)	8.22 (2.39)	3.51 (2.74)	t(49) = 9.49 ***	1.49 (L)
Sexual desire	4.53 (1.85)	7.42 (2.05)	2.89 (2.11)	t(49) = 10.18 ***	1.48 (L)
Intercourse satisfaction	8.58 (2.71)	10.67 (2.70)	2.10 (2.30)	t(49) = 6.75 ***	0.77 (M)
Overall Satisfaction	4.38 (1.95)	7.90 (2.10)	3.51 (2.39)	t(49) = 10.90 ***	1.74 (L)
Sexual function	37.29 (11.63)	55.65 (13.16)	18.64 (12.91)	t(49) = 10.54 ***	1.48 (L)
Pain scale	6.30 (2.45)	3.40 (2.12)	−2.90 (2.28)	t(49) = 8.24 ***	1.25 (L)
BMI (kg/m^2^)	22.5 (2.0)	22.3 (1.8)	−0.2 (0.5)	t(49) = 1.14	0.14 (S)
Relationship satisfaction	2.90 (0.75)	4.10 (0.83)	1.20 (1.02)	t(49) = 7.54 ***	1.45 (L)

SD: standard deviation. BMI: body mass index. *** *p* < 0.001. M = medium effect size. L = large effect size. S: short effect size.

## Data Availability

Data might be made available under the following conditions: (1) Whoever requests the data is an expert in the field and a senior researcher; (2) requests are made with the institutional email address (no@gmail.com or @hotmail.com or similar); (3) hypotheses are clearly formulated; (4) the expert in the field and the institution confirm and guarantee that all data are securely stored on a server of the institution that nobody else has access to; and (5) the scientist and the institution confirm and guarantee that data will not be shared with third parties, even when the third party belongs to the same institution.

## Abbreviations

LSS	lumbar spinal stenosis
IIEF	International Index of Erectile Function
